# A randomized, seven-day study to assess the efficacy and safety of a glycopyrrolate/formoterol fumarate fixed-dose combination metered dose inhaler using novel Co-Suspension™ Delivery Technology in patients with moderate-to-very severe chronic obstructive pulmonary disease

**DOI:** 10.1186/s12931-016-0491-8

**Published:** 2017-01-06

**Authors:** Colin Reisner, Leonardo M. Fabbri, Edward M. Kerwin, Charles Fogarty, Selwyn Spangenthal, Klaus F. Rabe, Gary T. Ferguson, Fernando J. Martinez, James F. Donohue, Patrick Darken, Earl St. Rose, Chad Orevillo, Shannon Strom, Tracy Fischer, Michael Golden, Sarvajna Dwivedi

**Affiliations:** 1 Pearl Therapeutics Inc., 280 Headquarters Plaza, East Tower, 2nd Floor, Morristown, NJ 07960 USA; 2 Department of Medicine, University of Modena and Reggio Emilia, NOCSAE, Modena, Italy; 3 Clinical Research Institute of Southern Oregon, Medford, OR USA; 4 Spartanburg Medical Research, Spartanburg, NC USA; 5 American Health Research, Charlotte, NC USA; 6 Lungen Clinic Grosshansdorf, Airway Research Center North, Member of the German Center for Lung Research (DZL), Grosshansdorf, Germany; 7 Department of Medicine, Christian-Albrechts University Kiel, Kiel, MI USA; 8 Pulmonary Research Institute of Southeast Michigan, Farmington Hills, MI USA; 9 Joan and Sanford I. Weill Department of Medicine, Weill Cornell Medical College; New York-Presbyterian Hospital/Weill Cornell Medical Center, New York, NY USA; 10 Department of Medicine, University of North Carolina School of Medicine, Chapel Hill, NC USA; 11 Pearl Therapeutics Inc., Durham, NC USA; 12 Pearl Therapeutics Inc., Redwood City, CA USA

**Keywords:** COPD, LAMA, LABA, Lung function, Bronchodilators, COPD maintenance, Co-Suspension™ Delivery Technology, Metered dose inhaler

## Abstract

**Background:**

Long-acting muscarinic antagonist/long-acting β_2_-agonist combinations are recommended for patients whose chronic obstructive pulmonary disease (COPD) is not managed with monotherapy. We assessed the efficacy and safety of glycopyrrolate (GP)/formoterol fumarate (FF) fixed-dose combination delivered via a Co-Suspension™ Delivery Technology-based metered dose inhaler (MDI) (GFF MDI).

**Methods:**

This was a Phase IIb randomized, multicenter, placebo-controlled, double-blind, chronic-dosing (7 days), crossover study in patients with moderate-to-very severe COPD (NCT01085045). Treatments included GFF MDI twice daily (BID) (GP/FF 72/9.6 μg or 36/9.6 μg), GP MDI 36 μg BID, FF MDI 7.2 and 9.6 μg BID, placebo MDI, and open-label formoterol dry powder inhaler (FF DPI) 12 μg BID or tiotropium DPI 18 μg once daily. The primary endpoint was forced expiratory volume in 1 s area under the curve from 0 to 12 h (FEV_1_ AUC_0–12_) on Day 7 relative to baseline FEV_1_. Secondary endpoints included pharmacokinetics and safety.

**Results:**

GFF MDI 72/9.6 μg or 36/9.6 μg led to statistically significant improvements in FEV_1_ AUC_0–12_ after 7 days’ treatment versus monocomponent MDIs, placebo MDI, tiotropium, or FF DPI (*p* ≤ 0.0002). GFF MDI 36/9.6 μg was non-inferior to GFF MDI 72/9.6 μg and monocomponent MDIs were non-inferior to open-label comparators. Pharmacokinetic results showed glycopyrrolate and formoterol exposure were decreased following administration via fixed-dose combination versus monocomponent MDIs; however, this was not clinically meaningful. GFF MDI was well tolerated.

**Conclusions:**

GFF MDI 72/9.6 μg and 36/9.6 μg BID improve lung function and are well tolerated in patients with moderate-to-very severe COPD.

**Trial registration:**

ClinicalTrials.gov NCT01085045. Registered 9 March 2010.

**Electronic supplementary material:**

The online version of this article (doi:10.1186/s12931-016-0491-8) contains supplementary material, which is available to authorized users.

## Background

Concurrent use of long-acting muscarinic antagonist (LAMA) and long-acting β_2_-agonist (LABA) therapy has been shown to maximize the bronchodilator response [1], and dual LAMA/LABA combination therapy is now recommended as an alternative option for patients whose chronic obstructive pulmonary disease (COPD) is not well managed with bronchodilator monotherapy [2, 3]. In addition to providing improvements in airflow limitation and symptom control, dual bronchodilator therapy reduces the risk of adverse effects that may be associated with increased dosages of a single bronchodilator [1]. The use of a fixed-dose combination (FDC), in which the two agents are combined in a single device, may improve adherence, leading to improved outcomes and reduced costs [4, 5].

There are currently options for both once-daily (QD) and twice-daily (BID) dosing of LAMA/LABA FDCs [6], with some evidence suggesting that BID dosing may be preferable for patients who experience night-time symptoms [7]. However, choice of delivery device has been limited to LAMA/LABA FDCs delivered by dry powder inhalers (DPIs) or Soft Mist™ devices. Thus there is an opportunity to expand patient choice by developing a metered dose inhaler (MDI) formulation-based LAMA/LABA FDC.

A Co-Suspension™ Delivery Technology has been developed to overcome the variability and instability associated with drug delivery via traditional hydrofluoroalkane propellant-based MDI devices. This technology uses a novel formulation technique in which active-agent particles (typically in the form of micronized drug crystals) form strong and non-specific associations within the propellant with specially engineered porous microparticles, made from distearoylphosphatidylcholine and calcium chloride. These formulations possess excellent stability and dose uniformity, and allow simultaneous delivery of multiple drugs from one MDI without one drug affecting the delivery of the others, and maintain consistency of dose and aerosol properties between monotherapies and their combinations [8].

This Phase IIb study investigated the efficacy and safety of a LAMA/LABA FDC MDI, GFF MDI, containing glycopyrrolate (GP; equivalent to the bromide salt, glycopyrronium bromide) and formoterol fumarate (FF) formulated using the Co-Suspension delivery technology, using the dose of glycopyrrolate identified in a dose-response study in patients with moderate-to-severe COPD [9]. Earlier Phase I/IIa studies evaluated the efficacy and safety of the individual components, using a previous formulation of phospholipid porous particles [10, 11].

The primary objective of this two-part study was to assess lung function, specifically the improvement in forced expiratory volume in 1 s (FEV_1_) area under curve (AUC) from 0 to 12 h post-dose (FEV_1_ AUC_0–12_). Firstly to assess improvements in FEV_1_ AUC_0–12_ with GFF MDI 72/9.6 μg or 36/9.6 μg BID compared with individual component MDIs, placebo MDI (suspension of porous microparticles only), or the open-label comparators FF DPI and tiotropium bromide DPI in patients with COPD. Secondly, to assess improvements in FEV_1_ AUC_0–12_ reported with FF 9.6 μg MDI versus placebo MDI in patients with COPD. The safety profiles of GFF MDI and FF MDI, as well as the pharmacokinetic (PK) profiles of both glycopyrrolate and formoterol after chronic administration of GFF MDI, were also investigated.

## Methods

### Patients

Patients were 40–80 years of age with a diagnosis of COPD and were current or former smokers, with a smoking history of at least 10 pack-years. Key lung function criteria were: pre- and post-bronchodilator FEV_1_/forced vital capacity (FVC) ratio <0.7; post-bronchodilator FEV_1_ ≥750 mL, and ≥30% and <80% of the predicted value at screening; and pre-bronchodilator FEV_1_ ≤ 80% at baseline. Key exclusion criteria were: pregnancy or lactation; respiratory disease other than COPD; poorly managed COPD that had required hospitalization within 3 months of screening, or treatment with corticosteroids or antibiotics within 6 weeks of screening. In addition, patients who did not meet American Thoracic Society criteria for acceptable spirometry were excluded. Patients provided informed consent before undergoing any screening assessments.

### Study design

This was a Phase IIb randomized, multicenter, placebo-controlled, double-blind, chronic dosing (7 days), four-period, eight-treatment, incomplete-block, crossover study, conducted in two parts in the USA, Australia, and New Zealand (NCT01085045). Patients recruited to Part A were not eligible for Part B. The design of the study is depicted in Additional file 1: Figure S1.

Part A was a four-period, eight-treatment, incomplete-block crossover study, designed to evaluate eight treatments: (i) GFF MDI 72/9.6 μg BID; (ii) GFF MDI 36/9.6 μg BID; (iii) GP MDI 36 μg BID; (iv) FF MDI 9.6 μg BID; (v) FF MDI 7.2 μg BID; (vi) placebo MDI BID; (vii) FF DPI 12 μg BID; and (viii) tiotropium DPI 18 μg QD. In this report, GP was expressed as glycopyrrolate (also known as glycopyrronium bromide) for which ex-actuator doses of 36 μg and 72 μg are equivalent to glycopyrronium (active moiety) 28.8 μg and 57.6 μg, respectively. Similarly, FF was expressed as formoterol fumarate, for which the dose of 9.6 μg (ex-actuator) is equivalent to formoterol fumarate dihydrate 10 μg. Each patient received four of eight possible treatments. A given treatment sequence included a GP MDI or an FF MDI component in no more than two treatment periods, whether administered as an FDC or as a single agent. Six combinations of four treatments were chosen for the study and then 48 treatment sequences created. Patients were randomized to one of the 48 treatment sequences which were generated centrally using an Interactive Web-based Response System based on Williams Square layouts.

Part B was a four-period, four-treatment, full-crossover study designed to evaluate: (i) FF MDI 9.6 μg BID; (ii) FF MDI 7.2 μg BID; (iii) placebo MDI BID; and (iv) FF DPI 12 μg BID. Patients were randomized to one of 24 possible treatment sequences, which were generated in the same way as Part A.

Patients administered each of their four assigned treatments for 1 week, followed by a 7- to 21-day washout period between treatments. All inhalers were dispensed in a blinded manner, with the exception of FF DPI and tiotropium DPI, which were provided open-label. The first dose of study drug was administered at the clinic under the supervision of a study coordinator (patients had been assessed previously for correct use of the MDI by study staff when using an albuterol MDI for the bronchodilator reversibility assessment). Self-administration continued at home. Each dose comprised two MDI actuations. Patients used their study drug BID for 1 week. FF DPI and open-label tiotropium DPI were administered for 7 days, according to the manufacturer’s instructions.

This study was conducted in accordance with International Conference on Harmonization guidelines, the Declaration of Helsinki and the US Code of Federal Regulations. The protocol, its amendments and patient informed consent form were approved by an Independent Ethics Committee or Institutional Review Board.

### Efficacy endpoints

In both parts of the study, the primary endpoint was FEV_1_ AUC_0–12_ on Day 7 relative to baseline FEV_1_. In Part A, there were two primary comparisons: (i) GFF MDI 72/9.6 μg BID versus GP MDI 36 μg BID and (ii) GFF MDI 72/9.6 μg BID versus FF MDI 9.6 μg BID. To demonstrate efficacy for GFF MDI, superiority to both monocomponent MDIs was required. In Part B, the primary endpoint was based on the comparison of FF MDI 9.6 μg BID with placebo MDI BID.

The secondary efficacy endpoints of the study included measurements on both Day 1 and Day 7: peak change from baseline in FEV_1_, time to onset of action (≥10% improvement in FEV_1_ relative to baseline), proportion of patients achieving ≥12% improvement in FEV_1_ relative to baseline, peak change from baseline in inspiratory capacity (IC) and change in morning pre-dose trough FEV_1_ and IC. An exploratory endpoint included change from baseline in FVC. These endpoints provided additional information on the dose-response of bronchodilator effects on lung function for GFF MDI and FF MDI by exploring two doses for each versus active comparators and placebo MDI: GFF MDI 72/9.6 μg and 36/9.6 μg, and FF MDI 7.2 μg and 9.6 μg.

### Pharmacokinetics

PK parameters were derived from the plasma concentrations of glycopyrrolate and formoterol fumarate obtained on approximately Day 7 (Day 7 ± 2) of each treatment regimen during Study Parts A and B. In Part A, the PK profiles of glycopyrrolate and formoterol after chronic administration of GFF MDI were compared with those after chronic administration of the monocomponent MDIs. In Part B, the PK profile of formoterol after chronic administration of two dose levels of FF MDI was compared with those after chronic administration of FF DPI. PK samples were collected at pre-dose, at 2, 6 and 20 min, and at 1, 2, 4, 8, 10 and 12 h post-dose. PK analyses were performed by Pharsight Inc using a validated version of WinNonlin^®^ Enterprise (Version 5.2).

### Safety evaluations

In addition to monitoring adverse events (AEs) and serious AEs (SAEs), the following safety evaluations were performed: 12-lead electrocardiogram (ECG), vital signs, physical examination, clinical laboratory tests, and evaluation for symptoms of AEs of interest including dry mouth, tremor and paradoxical bronchospasm.

### Statistical analysis

Data processing, data screening, descriptive reporting and analysis of the efficacy and safety data were performed using SAS Version 9.2 (SAS Institute, Inc., Cary, NC). The PK data were analyzed using WinNonLin Version 5.2 (Pharsight Corp., USA). PK graphs were prepared using SigmaPlot for Windows Version 9.01 (Systatsoftware, Inc., San Jose, CA). Power calculations were performed using R software. Further details of statistical methods and analysis are detailed in the Additional file 1.

The primary efficacy analysis was based on a modified intent-to treat (mITT) population, defined as patients who completed at least two treatment periods up to at least 2 h post-dose on Day 7 (with no more than one missing data point from the 15-min to the 2-h post-dose timepoint, inclusive); patients whose baseline FEV_1_ at Visits 4, 6 and 8 was not within 15% of baseline FEV_1_ at Visit 2 (reproducibility criteria) were also excluded from the mITT population. A separate population, PK-mITT, was defined for use in the PK analyses.

Data from Part A and Part B were combined for analysis using a linear mixed-effects model of the primary endpoint FEV_1_ AUC_0–12_ (baseline FEV_1_ was included as a covariate). The AUC was calculated using trapezoidal integration on the available timepoints. Superiority testing was performed using a two-sided 0.05 level of significance; non-inferiority testing was performed using a 0.025 level of significance based on a one-sided confidence interval (CI). The pre-defined non-inferiority margin for continuous spirometry variables was 100 mL, selected on the basis that it is the minimally clinically significant difference, defined as the change in FEV_1_ that can be perceived by the patient [12]. As such, non-inferiority was only confirmed for a treatment group if the relevant bound of the two-sided 95% CI for the difference was above −100 mL or below 100 mL. Mean changes from baseline in FEV_1_ were provided with 95% CI to support any conclusions of non-inferiority.

### Sample size calculations

Calculations to determine adequate sample size were based on the primary endpoint, FEV_1_ AUC_0–12_. For superiority testing of spirometry parameters, a difference of 100 mL was the pre-defined minimally clinically significant difference to be observed in FEV_1_ AUC_0–12_. Combined data from Part A and Part B gave the study the power of approximately 82–95% to detect the minimally clinically significant difference in FEV_1_ AUC_0–12_ between the treatment comparisons of interest.

### Role of the funding source

The funder of the study was involved in study design, data collection, data analysis, data interpretation and writing of the report. All authors had full access to all the data in the study and the corresponding author had the final responsibility for the decision to submit for publication. No restrictions were placed on authors regarding the statements made in the manuscript.

## Results

### Patient disposition

**Fig. 1:**
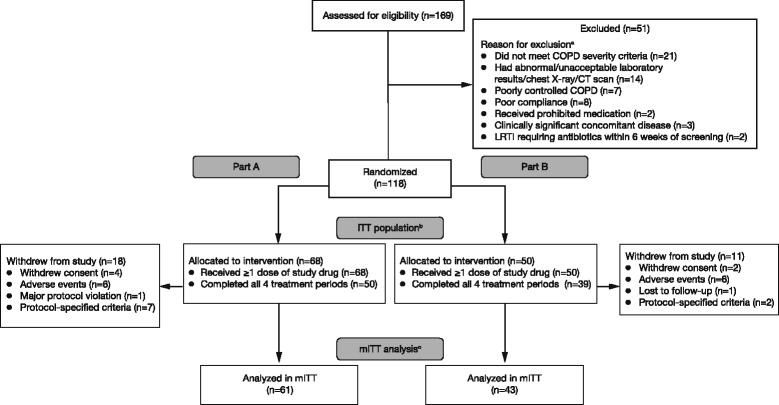
Patient disposition. In Part A, patients were randomized to receive any of the eight treatments in each of the four periods of the study in an incomplete block crossover design. In Part B, patients were randomized to receive all three formoterol doses and placebo in each of the four periods of the study in a full crossover design. ^a^Five patients met multiple criteria for exclusion from randomization (not meeting inclusion criteria and/or meeting exclusion criteria). ^b^Patients randomized to treatment, who received at least one dose of study drug. ^c^Patients who completed at least two treatment periods with at least 2 h of post-dose data on Day 7, with no more than one missing data-point from 15 min to 2 h post-dose, inclusive. COPD, chronic obstructive pulmonary disease; CT, computed tomography; ITT, intent-to-treat; LRTI, lower respiratory tract infection; mITT, modified ITT

A total of 169 patients were screened and 122 were randomized between 24 March 2010 and 28 October 2010 to receive treatment at sites in Australia, New Zealand and the USA. Following review of data from four sentinel patients, 118 patients were randomized: 68 patients into Part A and 50 patients into Part B (Fig. 1). All 118 patients received at least one dose of study drug and were included in the ITT population and 104 patients (88.1%) were included in the mITT population. Major reasons for exclusion from the mITT population were: (i) patient did not complete at least two treatment periods up to at least 2 h post-dose on Day 7 (Part A, 10.3% of patients and Part B, 14.0% of patients), and (ii) patient failed reproducibility criteria or had missing data on Day 7 (maximum of 3.4% patients in any one period of the study). There were 82 patients (69.5%) included in the per-protocol population (patients who completed all four treatment periods). The majority of patients (80.5 to 96.2% across treatment groups) received 80 to 100% of their assigned treatment regimen.

### Baseline characteristics

Patients’ baseline and demographic characteristics are shown in Table 1 (mITT population). Briefly, the mean (± standard deviation [SD]) duration of patients’ history of COPD was 7.7 (±5.9) years; the mean post-bronchodilator FEV_1_ was 50.8 (±12.7) % of predicted; and the mean FEV_1_ bronchodilator reversibility was 16.9 (±14.9) %. Overall, 52.9% (55/104) of patients had moderate COPD, 44.2% (46/104) had severe COPD and 2.9% (3/104) had very severe COPD.

**Table 1:** Baseline demographics (mITT population)

Parameter	GFF MDI		GP MDI 36 μg (*N* = 41)	Open-label tiotropium 18 μg (*N* = 58)	FF MDI		Placebo MDI (*N* = 52)	Open-label FF^a^ DPI 12 μg (*N* = 55)
	72/9.6 μg (*N* = 41)	36/9.6 μg (*N* = 43)			9.6 μg (*N* = 64)	7.2 μg (*N* = 64)		
Age, years								
Mean (SD)	62.4 (9.4)	63.3 (8.3)	66.3 (6.1)	64.1 (7.9)	63.4 (8.9)	63.6 (8.9)	62.8 (9.6)	60.6 (9.0)
Gender, *n* (%)								
Male	25 (61.0)	24 (55.8)	23 (56.1)	34 (58.6)	34 (53.1)	36 (56.3)	29 (55.8)	34 (61.8)
Race, *n* (%)								
Black/African	0	0	0	0	3 (4.7)	3 (4.7)	3 (5.8)	3 (5.5)
White	39 (95.1)	42 (97.7)	41 (100)	57 (98.3)	61 (95.3)	61 (95.3)	48 (92.3)	52 (94.5)
Australia/New Zealand (indigenous)	2 (4.9)	1 (2.3)	0	1 (1.7)	0	0	1 (1.9)	0
Smoking status, *n* (%)								
Current	16 (39.0)	18 (41.9)	15 (36.6)	24 (41.4)	29 (45.3)	28 (43.8)	24 (46.2)	25 (45.5)
Former	25 (61.0)	25 (58.1)	26 (63.4)	34 (58.6)	35 (54.7)	36 (56.3)	28 (53.8)	30 (54.5)
Duration of COPD, years								
Mean (SD)	7.6 (7.3)^b^	6.2 (5.4)^c^	7.8 (6.2)^b^	7.4 (6.7)^d^	8.6 (6.1)^e^	7.7 (4.4)^f^	8.3 (5.2)^g^	7.3 (4.3)^h^
Mean % predicted FEV_1_ (SD)								
Pre-bronchodilator	44.1 (13.9)^b^	46.8 (14.1)^c^	45.8 (13.5)^b^	44.9 (13.9)^d^	44.7 (12.6)^e^	43.9 (12.0)^f^	43.7 (11.6)^g^	44.0 (13.3)^h^
Post-bronchodilator	50.6 (13.0)^b^	53.0 (13.1)^c^	51.5 (13.3)^b^	51.3 (13.4)^d^	51.4 (12.5)^e^	50.2 (12.6)^f^	51.1 (12.4)^g^	50.9 (12.9)^h^
Mean FEV_1_, L (SD)								
Pre-bronchodilator	1.33 (0.48)^b^	1.38 (0.47)^c^	1.30 (0.41)^b^	1.33 (0.47)^d^	1.29 (0.43)^e^	1.28 (0.40)^f^	1.30 (0.41)^g^	1.35 (0.46)^h^
Post-bronchodilator	1.52 (0.47)^b^	1.56 (0.47)^c^	1.46 (0.40)^b^	1.51 (0.46)^d^	1.49 (0.46)^e^	1.47 (0.43)^f^	1.52 (0.47)^g^	1.56 (0.48)^h^
FEV_1_ bronchodilator reversibility, L (SD)^i^								
Mean (SD)	17.8 (16.3)^b^	16.3 (17.2)^c^	14.2 (14.5)^b^	17.1 (16.2)^d^	17.5 (14.7)^e^	15.9 (12.7)^f^	18.6 (12.9)^g^	18.5 (15.5)^h^

^a^Foradil^®^ Aerolizer^®^; ^b^ *n* = 38; ^c^ *n* = 39; ^d^ *n* = 56; ^e^ *n* = 58; ^f^ *n* = 63; ^g^ *n* = 45; ^h^ *n* = 54; ^i^percentage change from pre-albuterol at 30 min post-albuterol for FEV_1_

% = 100 × n/N, where *n* = number of patients in category and *N* = number of patients in the group

Duration of COPD = (date of first dose of study treatment in the study – COPD onset date)/365.25

Data from four sentinel patients were included in the mITT population in the analyses of demographic and baseline characteristics only

*COPD* chronic obstructive pulmonary disease, *DPI* dry powder inhaler, *FEV*_1_ forced expiratory volume in 1 s, *FF* formoterol fumarate, *GFF* glycopyrrolate/formoterol fumarate, *GP* glycopyrrolate, *MDI* metered dose inhaler, *mITT* modified intent-to-treat, *SD* standard deviation

### FEV_1_ AUC_0–12_ on Day 7

**Fig. 2:**
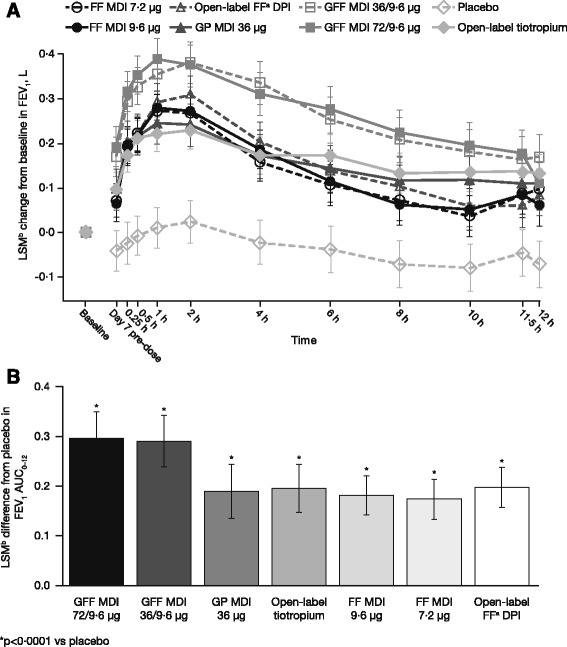
FEV_1_ AUC_0–12_ on Day 7 efficacy endpoint. **a** LSM change (95% CI) in FEV_1_ over 0–12 h on Day 7 by treatment; **b** LSM (95% CI) FEV_1_ AUC_0–12_ difference from placebo on Day 7 by treatment (mITT population). ^a^Foradil^®^ Aerolizer^®^^. b^LSM allows for any imbalances in baseline covariates that relate to responses to be adjusted for in order to avoid bias in treatment effect estimates. AUC_0–12_, area under the curve from 0 to 12 h post-dose; DPI, dry powder inhaler; FEV_1_, forced expiratory volume in 1 s; FF, formoterol fumarate; GFF, glycopyrrolate/formoterol fumarate; GP, glycopyrrolate; LSM, least squares mean; MDI, metered dose inhaler; mITT, modified intent-to-treat

Figure 2a shows the least squares mean (LSM) change from baseline in FEV_1_ over 12 h on Day 7. All active treatments were superior to placebo MDI for FEV_1_ AUC_0–12_ on Day 7 (*p* < 0.0001) (Figure 2b). GFF MDI 36/9.6 μg was non-inferior to GFF MDI 72/9.6 μg in FEV_1_ AUC_0–12_ on Day 7 since the upper bound of the CI was <100 mL (LSM difference between treatments = 0.008 L; 95% CI = −0.039, 0.054 L). GFF MDI 72/9.6 μg and GFF MDI 36/9.6 μg each demonstrated superior bronchodilation of 101 to 124 mL compared with their individual component MDIs, GP MDI 36 μg, FF MDI 9.6 μg and FF MDI 7.2 μg, as well as superior bronchodilation compared with the open-label comparators FF DPI and tiotropium DPI (*p* ≤ 0.0002) for FEV_1_ AUC_0–12_ on Day 7 (Table 2). GP MDI 36 μg, demonstrated non-inferiority to the LAMA comparator open-label tiotropium DPI for FEV_1_ AUC_0–12_ on Day 7 (LSM difference between treatments = −0.006 L; 95% CI = −0.049, 0.038 L). Both doses of FF MDI (7.2 and 9.6 μg) demonstrated non-inferiority to the open-label comparator FF DPI (Table 2).

**Table 2:** FEV_1_ AUC_0–12_ at Day 7: GFF MDI 72/9.6 μg and 36/9.6 μg comparisons (mITT population)

	LSM treatment differences for GFF MDI in FEV_1_ AUC_0–12_ at Day 7							
	GFF MDI		GP MDI 36 μg	Open-label tiotropium 18 μg	FF MDI		Placebo MDI	Open-label FF^a^ DPI 12 μg
Comparator	72/9.6 μg	36/9.6 μg			9.6 μg	7.2 μg		
GFF MDI 72/9.6 μg								
LSM^b^ difference (SE), L	NA	0.008 (0.0236)	0.109 (0.0250)^†^	0.103 (0.0216)^†^	0.116 (0.0245)^†^	0.124 (0.0237)^†^	0.298 (0.0261)^†^	0.101 (0.0241)^†^
95% CI		−0.039, 0.054	0.059, 0.158^c^	0.060, 0.145	0.068, 0.165	0.078, 0.171	0.247, 0.349	0.053, 0.148
GFF MDI 36/9.6 μg								
LSM^b^ difference (SE), L	See above	NA	0.101 (0.0245)^†^	0.095 (0.0213)^†^	0.109 (0.0242)^†^	0.116 (0.0236)^†^	0.290 (0.0261)^†^	0.093 (0.0241)^***^
95% CI			0.053, 0.149	0.053, 0.137	0.061, 0.156	0.070, 0.163	0.239, 0.342	0.045, 0.140

^***^ *p* < 0.001; ^†^ *p* < 0.0001

^a^Foradil^®^ Aerolizer^®^; ^b^LSM allows for any imbalances in baseline covariates that relate to responses to be adjusted for in order to avoid bias in treatment effect estimates; ^c^non-inferiority comparison

CI, confidence interval; DPI, dry powder inhaler; FEV_1_ AUC_0–12_, forced expiratory volume in 1 s area under the curve from 0 to 12 h post-dose; FF, formoterol fumarate; GFF, glycopyrrolate/formoterol fumarate; GP, glycopyrrolate; LSM, least squares mean; MDI, metered dose inhaler; mITT, modified intent-to-treat; NA, not available; SE, standard error

### Secondary endpoints

All active treatments were superior to placebo MDI for the lung function secondary endpoints (peak change from baseline in FEV_1_; time to onset of action on Day 1 [≥10% improvement in FEV_1_ relative to baseline]; peak change from baseline FEV_1_ and change from baseline in morning pre-dose trough FEV_1_ and 12-h post-dose trough FEV_1_; peak change from baseline in IC, change from baseline in morning pre-dose trough IC and 12-h post-dose trough IC; mean daily peak flow readings) on Days 1 and 7 (*p* ≤ 0.0056). The percentage of patients achieving ≥12% improvement in FEV_1_ was 86.8% (GFF MDI 72/9.6 μg), 87.2% (GFF MDI 36/9.6 μg), 73.7% (GP MDI 36 μg) 66.1% (open-label tiotropium DPI 18 μg), 84.5% (FF MDI 9.6 μg), 82.5% (FF MDI 7.2 μg), 40.0% (placebo MDI) and 85.2% (FF DPI 12 μg). Inferential comparisons of the percentage of patients achieving ≥12% improvement in FEV_1_ were not possible for several comparisons due to the limited number of patients (≤5) receiving each pair of treatments. However, numerical benefits were observed for all active treatments compared to placebo. GFF MDI 36/9.6 μg demonstrated non-inferiority to GFF MDI 72/9.6 μg (Table 3; Additional file 1). At Day 7, GFF MDI 72/9.6 μg and GFF MDI 36/9.6 μg demonstrated superiority to each monocomponent MDI and to open-label FF DPI and tiotropium for morning peak FEV_1_, peak change in FEV_1_, and morning pre-dose trough IC (Table 3; Additional file 1). FF MDI 7.2 μg and FF MDI 9.6 μg were both non-inferior to FF DPI, and FF MDI 7.2 μg was non-inferior to FF MDI 9.6 μg in secondary endpoints at Day 7 (Additional file 1).

**Table 3:** Secondary efficacy endpoints: Days 1 and 7 – GFF MDI 72/9.6 μg and GFF MDI 36/9.6 μg comparisons (mITT population)

Comparator	Treatment differences for GFF MDI comparisons					
	GP MDI 36 μg	Open-label tiotropium 18 μg	FF MDI		Placebo MDI	Open-label FF^a^ DPI 12 μg
			9.6 μg	7.2 μg		
DAY 7						
Change from baseline in morning pre-dose trough FEV_1_, L						
GFF MDI 72/9.6 μg						
LSM^b^ difference (SE)	0.0960 (0.0280)^***^	0.096 (0.0247)^†^	0.129 (0.0278)^†^	0.120 (0.0271)^†^	0.234 (0.0302)^†^	0.091 (0.0277)^**^
GFF MDI 36/9.6 μg						
LSM^b^ difference (SE)	0.073 (0.0273)^**^	0.073 (0.0245)^**^	0.106 (0.0274)^†^	0.097 (0.0270)^***^	0.211 (0.0300)^†^	0.068 (0.0275)^*^
Peak change from baseline in FEV_1_, L						
GFF MDI 72/9.6 μg						
LSM^b^ difference (SE)	0.125 (0.0282)^†^	0.140 (0.0248)^†^	0.101 (0.0279)^***^	0.108 (0.0271)^†^	0.342 (0.0300)^†^	0.082 (0.0278)^**^
GFF MDI 36/9.6 μg						
LSM^b^ difference (SE)	0.127 (0.0273)^†^	0.141 (0.0245)^†^	0.103 (0.0273)^***^	0.110 (0.0268)^†^	0.344 (0.0298)^†^	0.083 (0.0276)^**^
Change from baseline in morning pre-dose trough IC, L						
GFF MDI 72/9.6 μg						
LSM^b^ difference (SE)	0.083 (0.0445)	0.090 (0.0399)^*^	0.156 (0.0452)^***^	0.110 (0.0436)^*^	0.255 (0.0483)^†^	0.096 (0.0447)^*^
GFF MDI 36/9.6 μg						
LSM^b^ difference (SE)	0.098 (0.0445)^*^	0.105 (0.0387)^**^	0.172 (0.0433)^†^	0.126 (0.0428)^**^	0.271 (0.0471)^†^	0.111 (0.0434)^*^
Peak change from baseline in IC, L						
GFF MDI 72/9.6 μg						
LSM^b^ difference (SE)	0.078 (0.0532)	0.095 (0.0470)^*^	0.050 (0.0529)	0.033 (0.0513)	0.265 (0.0572)^†^	0.016 (0.0527)
GFF MDI 36/9.6 μg						
LSM^b^ difference (SE)	0.107 (0.0513)^*^	0.124 (0.0461)^**^	0.078 (0.0513)	0.062 (0.0503)	0.293 (0.0559)^†^	0.045 (0.0518)
DAY 1						
Peak change from baseline in FEV_1_, L						
GFF MDI 72/9.6 μg						
LSM^b^ difference (SE)	0.081 (0.0309)^**^	0.104 (0.0268)^†^	0.062 (0.0307)^*^	0.060 (0.0297)^*^	0.265 (0.0328)^†^	0.072 (0.0306)^*^
GFF MDI 36/9.6 μg						
LSM^b^ difference (SE)	0.068 (0.300)^*^	0.090 (0.0266)^***^	0.048 (0.0300)	0.046 (0.0293)	0.251 (0.0326)^†^	0.058 (0.0303)
Peak change from baseline in IC, L						
GFF MDI 72/9.6 μg						
LSM^b^ difference (SE)	0.065 (0.0567)	0.149 (0.0493)^**^	0.134 (0.0564)^*^	0.144 (0.0547)^**^	0.412 (0.0607)^†^	0.121 (0.0561)^*^
GFF MDI 36/9.6 μg						
LSM^b^ difference (SE)	−0.019 (0.0555)	0.065 (0.0491)	0.050 (0.0554)	0.060 (0.0542)	0.328 (0.0602)^†^	0.037 (0.0557)
Time to onset of action, hazard ratio^c^						
GFF MDI 72/9.6 μg						
HR	1.399^*^	1.754^***^	0.980	1.150	3.475^†^	0.971
95% CI	1.038, 1.884	1.300, 2.367	0.746, 1.289	0.904, 1.465	2.095, 5.765	0.713, 1.321
GFF MDI 36/9.6 μg						
HR	1.323	1.695^***^	0.888	1.062	3.358^†^	0.878
95% CI	0.936, 1.870	1.275, 2.253	0.671, 1.175	0.0806, 1.400	2.091, 5.391	0.660, 1.169

^*^ *p* < 0.05; ^**^ *p* < 0.01; ^***^ *p* < 0.001; ^†^ *p* ≤ 0.0001

^a^Foradil^®^ Aerolizer^®^; ^b^LSM allows for any imbalances in baseline covariates that relate to responses to be adjusted for in order to avoid bias in treatment effect estimates; ^c^a hazard ratio of 1.399 signifies a 39.9% higher probability of onset of action at any time point post-dose

*CI* confidence interval, *DPI* dry powder inhaler, *FEV*_1_ forced expiratory volume in 1 s, *FF* formoterol fumarate, *GFF* glycopyrrolate/formoterol fumarate, *GP* glycopyrrolate, *HR* hazard ratio, *IC* inspiratory capacity, *LSM* least squares mean, *MDI* metered dose inhaler, *mITT* modified intent-to-treat, *SE* standard error

### Exploratory endpoint

All active treatments were superior to placebo (*p* < 0.0001) for change from baseline FVC (calculated as AUC_0–12_) on Day 7 (Additional file 1: Figure S2). Treatment comparisons are shown in the Additional file 1.

### Safety and tolerability

Most AEs were of mild (32.0%) or moderate (29.5%) intensity. Treatment-emergent AEs (TEAEs) reported in more than two patients receiving treatment are displayed in Table 4. The most commonly reported TEAEs were: dry mouth, headache, tremor, cough and dysphonia (Table 4). Dry mouth was reported more frequently by patients receiving GP MDI, GFF MDI, and open-label tiotropium compared to the other groups, while headache and tremor were reported more frequently by patients receiving GFF MDI. No patient in any treatment period reported paradoxical bronchospasm. The incidence of TEAEs was similar for the two doses of GFF MDI (31.7% vs 27.9%).

**Table 4:** Summary of adverse events (safety population)

	GFF MDI		GP MDI 36 μg (*N* = 41)	Open-label tiotropium 18 μg (*N* = 58)	FF MDI		Placebo MDI (*N* = 52)	Open-label FF^a^ DPI 12 μg (*N* = 55)
	72/9.6 μg (*N* = 41)	36/9.6 μg (*N* = 43)			9.6 μg (*N* = 64)	7.2 μg (*N* = 64)		
Patients with at least one AE, *n* (%)	17 (41.5)	18 (41.9)	11 (26.8)	22 (37.9)	24 (37.5)	16 (25.0)	9 (17.3)	17 (30.9)
Patients with AE related to study treatment, *n* (%)	13 (31.7)	12 (27.9)	7 (17.1)	7 (12.1)	7 (10.9)	4 (6.3)	2 (3.8)	7 (12.7)
Patients with SAE, *n* (%)	0	1 (2.3)	0	2 (3.4)	1 (1.6)	2 (3.1)	0	0
Patients with SAE related to study treatment, *n* (%)	0	0	0	0	0	0	0	0
Patients with AE leading to early withdrawal, *n* (%)	1 (2.4)	0	1 (2.4)	1 (1.7)	4 (6.3)	3 (4.7)	1 (1.9)	0
Patients with SAE leading to early withdrawal, *n* (%)	0	0	0	0	0	2 (3.1)	0	0
TEAEs reported in ≥2 patients in any treatment group								
Dry mouth	8 (19.5)	3 (7.0)	5 (12.2)	4 (6.9)	3 (4.7)	2 (3.1)	1 (1.9)	2 (3.6)
Headache	3 (7.3)	4 (9.3)	1 (2.4)	1 (1.7)	1 (1.6)	0	1 (1.9)	2 (3.6)
Tremor	1 (2.4)	5 (11.6)	0	0	0	0	0	0
Cough	0	2 (4.7)	0	1 (1.7)	0	0	0	0
Dysphonia	1 (2.4)	2 (4.7)	0	0	0	0	0	0

% = 100 × n/N: *n* = no. of patients in the preferred term category for treatment group

^a^Foradil^®^ Aerolizer^®^

*AE* adverse event, *DPI* dry powder inhaler, *FF* formoterol fumarate, *GFF* glycopyrrolate/formoterol fumarate, *GP* glycopyrrolate, *MDI* metered dose inhaler, *SAE* serious adverse event, *TEAE* treatment-emergent adverse event

Six SAEs were reported in five patients, none of which was related to study drug (one patient in the GFF MDI 36/9.6 μg group [ruptured appendix]; two patients in the open-label tiotropium group [inhaled foreign body; abdominal aortic aneurysm]; one patient in the FF MDI 9.6 μg group [gastritis]; and two patients in the FF MDI 7.2 μg group [COPD exacerbation; atypical chest pain leading to early withdrawal]). No deaths were reported in the study.

There were no notable changes in hematology or chemistry laboratory values and no clinically significant abnormalities in vital signs, ECG, or physical examination.

### Pharmacokinetics

**Fig. 3:**
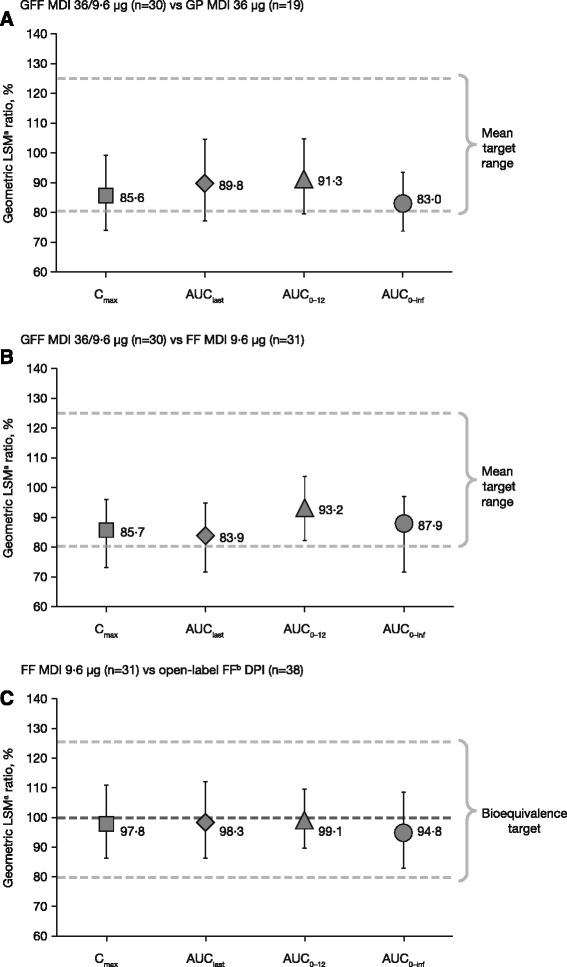
Ratio of geometric LSMs and 90% CIs. **a** GFF MDI 36/9.6 μg versus GP MDI 36 μg (**b**) GFF MDI 36/9.6 μg versus FF MDI 9.6 μg (**c**) FF MDI 9.6 μg versus FF DPI (PK-mITT population). ^a^LSM allows for any imbalances in baseline covariates that relate to responses to be adjusted for in order to avoid bias in treatment effect estimates. ^b^Foradil^®^ Aerolizer^®^^.^ AUC_0–inf_, area under the curve from time 0 to infinity; AUC_0–12_, area under the curve from 0 to 12 h post-dose; CI, confidence interval; C_max_, maximum observed plasma concentration; DPI, dry powder inhaler; FF, formoterol fumarate; GFF, glycopyrrolate/formoterol fumarate; GP, glycopyrrolate; LSM, least squares mean; MDI, metered dose inhaler; PK-mITT, pharmacokinetic modified intent-to-treat

Following chronic administration of GFF MDI 36/9.6 μg, the geometric LSM of glycopyrrolate was approximately 9% (AUC_0–12_) and 14% (maximum observed plasma concentration [C_max_]) lower than those observed following GP MDI 36 μg (Fig. 3a). In addition, the geometric LSM for formoterol was approximately 7% (AUC_0–12_) and 14% (C_max_) lower than those observed when FF MDI 9.6 μg was administered alone (Fig. 3b).

Results from an analysis of variance (ANOVA) of the dose-normalized exposure parameters of formoterol between the FF MDI 9.6 μg and the FF DPI showed that 90% CIs for the ratios of LSM for the exposure parameters AUC_0–12_ and C_max_ were within the 80–125% interval, demonstrating that monocomponent FF MDI 9.6 μg was bioequivalent to the FF DPI formulation (equivalent to an FF 10 μg dose), with dose normalization. Furthermore, ANOVA results based on non-dose-normalized PK parameters also demonstrated equivalence between FF MDI and FF DPI (Fig. 3c).

## Discussion

This 7-day Phase IIb study of GFF MDI 72/9.6 μg and 36/9.6 μg BID is the first study to investigate the FDC GFF MDI formulated using Co-Suspension delivery technology in patients with COPD. Previous studies have investigated the dose-response of the individual components delivered via MDI using Co-Suspension delivery technology [9, 13]. GFF MDI 72/9.6 μg and 36/9.6 μg BID led to statistically significant and clinically relevant improvement in the primary endpoint FEV_1_ AUC_0–12_ at Day 7 compared with the monocomponent MDIs and placebo MDI in patients with moderate-to-very severe COPD and were well tolerated (*p* ≤ 0.0001).

It has become established that the combination of a LAMA and LABA provides benefits in lung function in patients with COPD over LAMA or LABA monotherapy in those patients who are not adequately controlled by a single long-acting bronchodilator. Within this therapeutic approach, LAMA/LABA FDCs glycopyrrolate/indacaterol, tiotropium/olodaterol, aclidinium/formoterol, and umeclidinium/vilanterol have previously demonstrated lung function benefits over monocomponents [14–19]. This study was part of a Phase IIb program including dose-ranging studies of GFF MDI and monocomponents to define the optimal doses of the GFF MDI FDC to take forward to Phase III studies, and utilized non-inferiority testing, based on the targeted 100 mL minimally clinically significant difference, as a well-defined and reproducible treatment effect for trough FEV_1_ to define therapeutic effect [12]. In this study, non-inferiority was confirmed between GFF MDI 72/9.6 μg and 36/9.6 μg, incorporating GP doses at the higher end of the dose range, and both doses showed statistically significantly greater FEV_1_ AUC_0–12_ at Day 7 versus open-label tiotropium DPI and FF DPI. Notably, GFF MDI demonstrated superiority to placebo and statistically significant (GFF MDI 72/9.6 μg) and numerically greater (GFF MDI 36/9.6 μg) improvements in IC compared with open-label tiotropium DPI. In patients with COPD, changes in IC reflect changes in hyperinflation and have shown a higher correlation to patient-focused outcomes, such as dyspnea with exercise, than other standard spirometric measurements [20]. Additional information was gained concerning the monocomponents, whereby the doses of the two monocomponents, GP and FF, demonstrated non-inferiority to the open-label active comparators such that GP MDI 36 μg BID was non-inferior to open-label tiotropium DPI and both doses of FF MDI demonstrated non-inferiority to open-label FF delivered via DPI.

The PK component of the study characterized the systemic exposure of glycopyrrolate and formoterol delivered as an FDC compared with individual components delivered using Co-Suspension delivery technology. The findings support the absence of a significant drug-drug interaction for formoterol and glycopyrrolate following administration of GFF MDI relative to the individual MDI formulations. It was also shown that formoterol exposure (AUC_0–12_) increases in a dose-proportional manner when delivered via the MDI and that FF MDI 9.6 μg delivered by MDI using Co-Suspension delivery technology was bioequivalent to FF delivered using DPI, which taken together endorse the dose of FF MDI 9.6 μg in the FDC.

All treatments were generally well tolerated in this study. The tolerability and safety profiles observed for GFF MDI 36/9.6 μg and 72/9.6 μg BID were consistent with the patient population and drug classes [21]. The most commonly reported TEAEs (more than two patients in any treatment group) were dry mouth, headache, tremor, cough and dysphonia in descending order of incidence.

Whilst the sample size of this first study of GFF delivered by MDI using novel Co-Suspension delivery technology was calculated to provide reasonable information to characterize the response, we recognize that the study was limited by a relatively small population and enrolled a lower than anticipated number of patients with very severe COPD. In addition, the study was only conducted over 7 days. However, information from this study guided study design and sample size for subsequent studies.

GFF MDI will potentially widen treatment options for patients with COPD by providing LAMA/LABA therapy in an MDI when other LAMA/LABA FDCs are available as DPI and soft-mist inhaler devices. Unlike other devices, MDIs are not breath-actuated, consequently, MDIs may be able to offer patients with quite severe airflow limitation, who may be unable to breathe deeply enough to release the medication from a DPI, a more practical and reliable method of delivery of their medication. The PKs of GFF MDI support twice-daily administration and the improvements in lung function demonstrated here after BID dosing with GFF MDI as maintenance dual-bronchodilator therapy may provide benefits over QD dosing, preventing excessive worsening of symptoms during the night or towards the morning in patients with this pattern of symptoms [7]. The results of a patient evaluation survey conducted by Partridge et al., and similarly in the ASSESS study, show that patients with COPD begin to experience worsening symptoms in the evening through the night-time, with the worst symptoms, affecting activity and productivity, occurring in the morning [22, 23]. This is also an area for future investigation of the effects of GFF MDI on COPD symptom burden.

## Conclusions

Co-Suspension delivery technology allows formulation of FDCs of different drug classes, at different concentrations, in a single MDI. GFF MDI 72/9.6 μg and 36/9.6 μg BID were associated with a similarly greater magnitude of effect on FEV_1_ AUC_0–12_ at Day 7 compared with the monocomponent MDIs and placebo MDI in patients with moderate-to-very severe COPD, which is likely to be the maximal therapeutic effect. Additional studies are required to establish the optimal doses of GP and FF for combination in the FDC GFF MDI.

## Additional file

Additional file 1: Study design; Additional Fig. 1. Study design schematic; Additional Fig. 2. Mean change from baseline in PEFR over time by treatment on Day 7 (mITT population); Additional Fig. 3. Mean change from baseline FVC over time by treatment on Day 7 (mITT population); Additional Table 1. Secondary efficacy endpoints: Days 1 and 7 – FF 9.6 μg and FF 7.2 μg comparisons (mITT population). (DOCX 466 kb)

## Abbreviations

AEs: Adverse events
AUC: Area under curve
BID: Twice daily
CI: Confidence interval
COPD: Chronic obstructive pulmonary disease
CT: Computed tomography
DPI: Dry powder inhaler
ECG: Electrocardiogram
FDC: Fixed-dose combination
FEV_1_ AUC_0–12_: Forced expiratory volume in 1 s area under the curve from 0 to 12 h
FEV_1_: Forced expiratory volume in 1 s
FF: Formoterol fumarate
FVC: Forced vital capacity
GFF MDI: Glycopyrrolate/formoterol fumarate MDI
GP: Glycopyrrolate
HFA: Hydrofluoroalkane
HR: Hazard ratio
IC: Inspiratory capacity
ITT: Intent-to-treat
LABA: Long-acting beta agonist
LAMA: Long-acting muscarinic antagonist
LRTI: Lower respiratory tract infection
LSM: Least squares mean
MDI: Metered dose inhaler
mITT: Modified intent-to treat
NA: Not available
PK: Pharmacokinetic
QD: Once-daily
SAEs: Serious adverse events
SD: Standard deviation
SE: Standard error
TEAEs: Treatment-emergent AEs
