# Erratum to: A randomized, seven-day study to assess the efficacy and safety of a glycopyrrolate/formoterol fumarate fixed-dose combination metered dose inhaler using novel Co-Suspension™ Delivery Technology in patients with moderate-to-very severe chronic obstructive pulmonary disease

**DOI:** 10.1186/s12931-017-0638-2

**Published:** 2017-08-21

**Authors:** Colin Reisner, Leonardo M. Fabbri, Edward M. Kerwin, Charles Fogarty, Selwyn Spangenthal, Klaus F. Rabe, Gary T. Ferguson, Fernando J. Martinez, James F. Donohue, Patrick Darken, Earl St. Rose, Chad Orevillo, Shannon Strom, Tracy Fischer, Michael Golden, Sarvajna Dwivedi

**Affiliations:** 1 Pearl Therapeutics Inc., 280 Headquarters Plaza, East Tower, 2nd Floor, Morristown, NJ 07960 USA; 2 Department of Medicine, University of Modena and Reggio Emilia, NOCSAE, Modena, Italy; 3 Clinical Research Institute of Southern Oregon, Medford, OR USA; 4 Spartanburg Medical Research, Spartanburg, NC USA; 5 American Health Research, Charlotte, NC USA; 6 Lungen Clinic Grosshansdorf, Airway Research Center North, Member of the German Center for Lung Research (DZL), Grosshansdorf, Germany; 7 Department of Medicine, Christian-Albrechts University Kiel, Kiel, MI USA; 8 Pulmonary Research Institute of Southeast Michigan, Farmington Hills, MI USA; 9 Joan and Sanford I. Weill Department of Medicine, Weill Cornell Medical College; New York-Presbyterian Hospital/Weill Cornell MedicalCenter, New York, NY USA; 10 Department of Medicine, University of North Carolina School of Medicine, Chapel Hill, NC USA; 11 Pearl Therapeutics Inc., Durham, NC USA; 12 Pearl Therapeutics Inc., Redwood City, CA USA

## Erratum

Upon Publication of the original article [1] several discrepancies were highlighted in the following sections; Results, Table 1, Table 2 and Fig. 3. These errors have since been acknowledged and corrected in this erratum

**The first sentence in the subsection “Baseline characteristics” originally read:** Patients’ baseline and demographic characteristics are shown in Table 1 (mITT population).

**This should read:** Patients’ baseline and demographic characteristics are shown in Table 1.

**The header of Table 1 read:** Baseline demographics (mITT population)

**This should read as:** Baseline demographics (ITT population)

It was noticed that Table 2 contained an error. In Table 2, data row 2, the footnote symbol ‘c’ was erroneously included in the third column. The footnote symbol ‘c ‘should be placed in the second column in this row. The corrected Table 2 is shown below.

**Table 2:** FEV_1_ AUC_0–12_ at Day 7: GFF MDI 72/9.6 μg and 36/9.6 μg comparisons (mITT population)

	LSM treatment differences for GFF MDI in FEV_1_ AUC_0–12_ at Day 7							
	GFF MDI		GP MDI 36 μg	Open-label tiotropium 18 μg	FF MDI		Placebo MDI	Open-label FF^a^ DPI 12 μg
Comparator	72/9.6 μg	36/9.6 μg			9.6 μg	7.2 μg		
GFF MDI 72/9.6 μg								
LSM^b^ difference (SE), L 95% CI	NA	0.008 (0.0236)–0.039, 0.054^c^	0.109 (0.0250)^†^0.059, 0.158	0.103 (0.0216)^†^0.060, 0.145	0.116 (0.0245)^†^0.068, 0.165	0.124 (0.0237)^†^0.078, 0.171	0.298 (0.0261)^†^0.247, 0.349	0.101 (0.0241)^†^0.053, 0.148
GFF MDI 36/9.6 μg								
LSM^b^ difference (SE), L 95% CI	See above	NA	0.101 (0.0245)^†^0.053, 0.149	0.095 (0.0213)^†^0.053, 0.137	0.109 (0.0242)^†^0.061, 0.156	0.116 (0.0236)^†^0.070, 0.163	0.290 (0.0261)^†^0.239, 0.342	0.093 (0.0241)^***^0.045, 0.140

^***^ *p <* 0.001; ^†^ *p <* 0.0001

^a^Foradil® Aerolizer®; ^b^LSM allows for any imbalances in baseline covariates that relate to responses to be adjusted for in order to avoid bias in treatment effect estimates; ^c^non-inferiority comparison

CI, confidence interval; DPI, dry powder inhaler; FEV_1_ AUC_0–12_, forced expiratory volume in 1 s area under the curve from 0 to 12 h post-dose; FF, formoterol fumarate; GFF, glycopyrrolate/formoterol fumarate; GP, glycopyrrolate; LSM, least squares mean; MDI, metered dose inhaler; mITT, modified intent-to-treat; NA, not available; SE, standard error

It was noticed that in Fig. 3b, the error bar on the 4th data point (AUC_inf_) was incorrectly given. The error bar on the 4th data point (AUCinf) should range from 74.88, to 103.28 The corrected Fig. 3b is shown below.

**Fig. 3:**
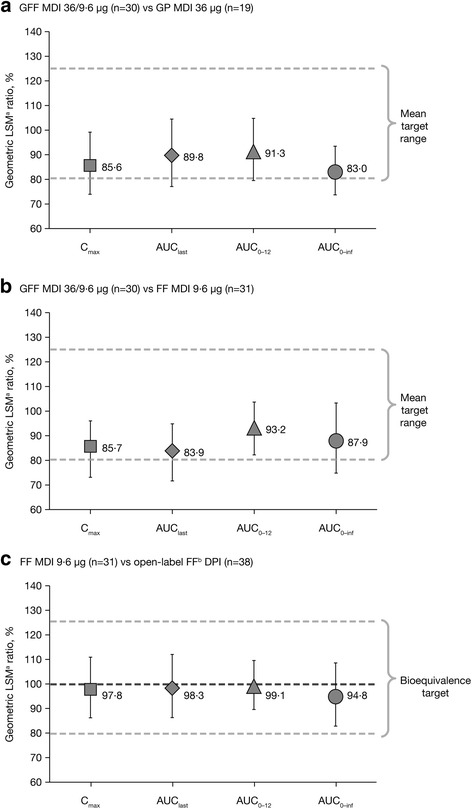
Ratio of geometric LSMs and 90% CIs. (**a**) GFF MDI 36/9.6 μg versus GP MDI 36 μg (**b**) GFF MDI 36/9.6 μg versus FF MDI 9.6 μg (**c**) FF MDI 9.6 μg versus FF DPI (PK-mITT population). ^a^LSM allows for any imbalances in baseline covariates that relate to responses to be adjusted for in order to avoid bias in treatment effect estimates. ^b^Foradil® Aerolizer®^.^AUC_0–inf_, area under the curve from time 0 to infinity; AUC_0–12_, area under the curve from 0 to 12 h post-dose; CI, confidence interval; C_max_, maximum observed plasma concentration; DPI, dry powder inhaler; FF, formoterol fumarate; GFF, glycopyrrolate/formoterol fumarate; GP, glycopyrrolate; LSM, least squares mean; MDI, metered dose inhaler; PK-mITT, pharmacokinetic modified intent-to-treat

These corrections do not change the conclusions of the article.
