# Supplementary material for: A randomized, seven-day study to assess the efficacy and safety of a glycopyrrolate/formoterol fumarate fixed-dose combination metered dose inhaler using novel Co-Suspension™ Delivery Technology in patients with moderate-to-very severe chronic obstructive pulmonary disease
Source: Respir Res. 2017 Jan 6;18:8. doi: 10.1186/s12931-016-0491-8 (PMC5216561; doi:10.1186/s12931-016-0491-8)
Supplement: Additional file 1: — Study design; Additional Fig. 1. Study design schematic; Additional Fig. 2. Mean change from baseline in PEFR over time by treatment on Day 7 (mITT population); Additional Fig. 3. Mean change from baseline FVC over time by treatment on Day 7 (mITT population); Additional Table 1. Secondary efficacy endpoints: Days 1 and 7 – FF 9.6 μg and FF 7.2 μg comparisons (mITT population). (DOCX 466 kb) [file 12931_2016_491_MOESM1_ESM.docx]

# ADDITIONAL MATERIALS

**Study design**

As this was the first chronic-dosing study conducted with the investigational treatments, four ‘sentinel patients’ (one each of GFF MDI 72/9.6 μg, GFF MDI 36/9.6 μg, GP MDI 36 μg and FF MDI 9.6 μg) were enrolled to provide information on, and additional assurance of, safety. After the safety results for sentinel patients had been evaluated by the Principal Investigator(s) in consultation with the sponsor’s Chief Medical Officer and were deemed acceptable, patients recruited to Part A were randomized to a treatment sequence by an interactive web-based response system.

The following medications were prohibited during the study: oral β2-agonists, LABAs, inhaled corticosteroid (ICS)/LABA combinations, theophylline, cromoglycate, nedocromil, leukotriene antagonists, tiotropium and oral corticosteroids. Patients who had been receiving maintenance ICS could continue such therapy throughout the duration of the study, if they been receiving stable dose of ICS for at least 4 weeks. Albuterol sulfate (salbutamol) inhalation aerosol was used to relieve symptoms of COPD as needed during the 7-day treatment period. Albuterol, ipratropium and albuterol/ipratropium combinations as MDIs were dispensed at the discretion of the investigator for use during the washout period between treatments.

**Assessments**

Spirometry: On Day 1 of each treatment period, spirometry was conducted 60 minutes and 30 minutes before study drug administration. The average of these two assessments was used to establish test-day baseline forced expiratory volume in 1 second (FEV_1_), and forced vital capacity (FVC). The baseline FEV_1_ result at Visits 4, 6 and 8 had to be within ±15 % of the baseline FEV_1_ obtained at the Randomization Visit/Visit 2. On Day 7 of each treatment period, spirometry was conducted 60 minutes and 30 minutes prior to study drug administration. The average of these two assessments was used to establish test-day baseline FEV_1_, and FVC. Following study drug administration, spirometry was obtained at 15 and 30 minutes and 1, 2, 4, 6, 8, 10, 11.5 and 12 hours post-dose.

Inspiratory capacity (IC) assessments: On Day 1 of each treatment period, IC assessments preceded spirometry and were obtained 60 minutes and 30 minutes pre-dose, and at 1 hour and 2 hours post-dose. On Day 7 of each treatment period, IC assessments were obtained at 60 minutes and 30 minutes pre-dose and at 1, 2, 11.5 and 12 hours post-dose.

Patient diary: Patients completed a daily dairy with study drug dosing time, their rescue medication use and peak flow measurements, and returned their diary at the next scheduled visit. The study coordinator reviewed the diary for completeness and accuracy,

**Pharmacokinetics**

Blood samples for pharmacokinetic (PK) analysis were collected pre-dose on Day 1 at Visits 2, 4, 6 and 8, and pre-dose and post-dose on Day 7 at Visits 3, 5, 7 and 9.

***Statistical analyses***

The PK-mITT population included only patients who remained in the study for at least 2 hours post-dose and provided sufficient and valid plasma drug concentration data, whether or not the patient completed all treatments. In addition, patients must have received at least one dose of a GP formulation (GFF MDI or GP MDI) and at least one dose of a FF formulation (GFF MDI, FF MDI, or Foradil® Aerolizer®). Data from sentinel patients were included in the ITT population (patients who received at least one dose of study drug) for the analyses of demographic and baseline characteristics only and in safety analyses.

An analysis of variance (ANOVA) was used in the analyses of the ln-transformed PK parameters area under the curve from time 0 to affinity (AUC)_inf_, AUC from 0 to 12 hours post-dose (AUC_0–12_) and maximum observed plasma concentration (C_max_) for glycopyrrolate and formoterol. A linear mixed model was used, which included fixed effects for treatment and period, with subject as a random factor.

**Additional Fig. 1. Study design schematic**

**
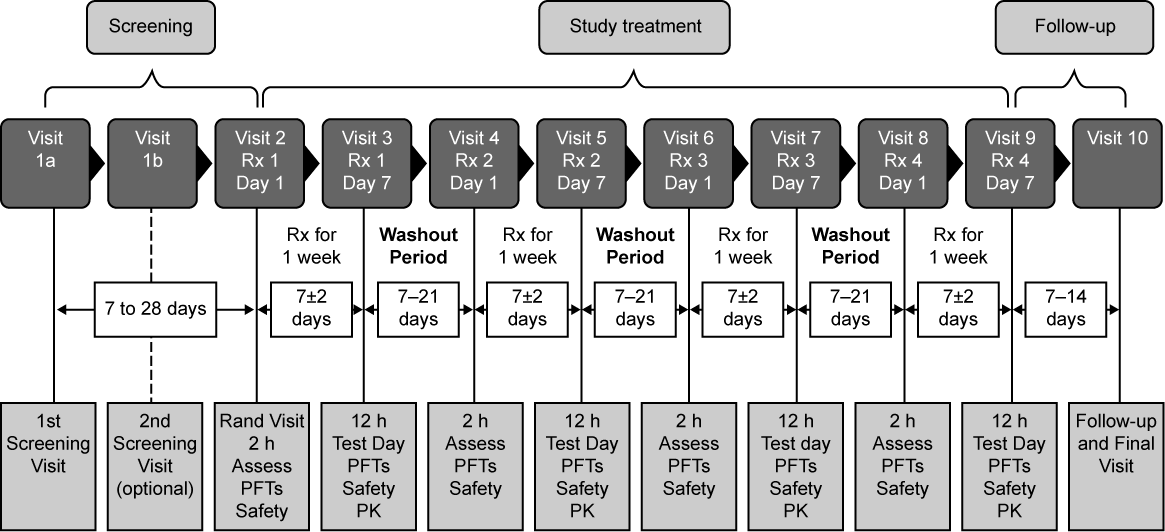
**

All sentinel patients completed Visits 1, 2, 3 and 10 study procedures (with the exception of PK assessments)

Assess, assessments; h, hour(s); PFT, pulmonary function test; PK, pharmacokinetic assessments; Rand, randomization; Rx, treatment

**Additional Fig. 2. Mean change from baseline FVC over time by treatment on Day 7 (mITT population)**


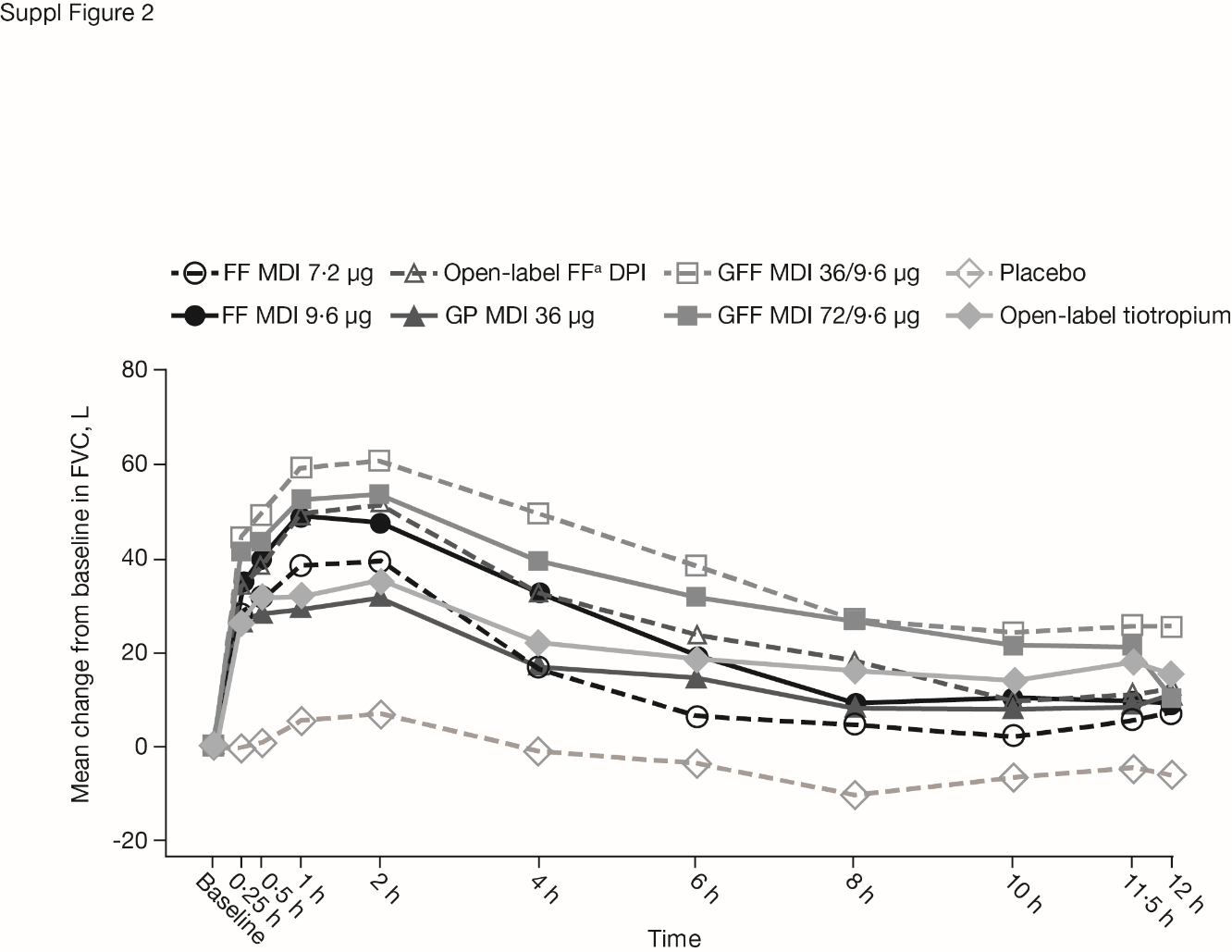


^a^Foradil^®^ Aerolizer^®^

DPI, dry powder inhaler; FF, formoterol fumarate; FVC, forced vital capacity; GFF, glycopyrrolate/formoterol fumarate; GP, glycopyrrolate; MDI, metered dose inhaler; mITT, modified intent-to-treat

**Additional Table 1. Secondary efficacy endpoints: Days 1 and 7 – FF 9.6 μg and FF 7.2 μg comparisons (mITT population)**

| **Treatment differences for FF MDI comparisons** | | | | | | |
| --- | --- | --- | --- | --- | --- | --- |
|  | **FF MDI 9.6 µg vs  FF MDI 7.2 µg** | **FF MDI 7.2 µg vs  FF MDI 9.6 µg** | **FF MDI 9.6 µg vs  Placebo MDI** | **FF MDI 7.2 µg vs  Placebo MDI** | **FF MDI 9.6 µg vs  FF^a^ DPI** | **FF MDI 7.2 µg vs  FF^a^ DPI** |
| **Day 1** | | | | | | |
| **Peak change from baseline in FEV_1,_ L** | | | | | | |
| LSM (SE)  95 % CI | –0.002 (0.0238) –0.049, 0.045 |  | 0.203 (0.0253) ^†^ 0.153, 0.253 | 0.205 (0.0253) ^†^ 0.155, 0.255 | 0.010 (0.0245)  –0.038, 0.058 | 0.012 (0.0242) –0.036, 0.059 |
| **Time to onset of action (≥10 % improvement) in FEV_1_** | | | | | | |
| HR  95 % CI | 1.164 0.921, 1.471 | 0.859 0.680, 1.085 | 3.467^†^ 2.161, 5.561 | 2.912^†^ 1.893, 4.478 | 0.988 0.773, 1.261 | 0.830 0.649, 1.061 |
| **Percentage of patients achieving ≥12 % improvement in FEV_1_** | | | | | | |
| % difference | 0.00 | 0.00 | 47.37^†^ | 48.72^†^ | –2.56 | –5.13 |
| **Day 7** | | | | | | |
| **Peak change from baseline in FEV_1_** | | | | | | |
| LSM (SE)  95 % CI | 0.007 (0.0218) –0.036, 0.050 |  | 0.241 (0.0232)^†^ 0.195, 0.286 | 0.234 (0.0234)^†^ 0.188, 0.280 | –0.019 (0.0223) –0.063, 0.025 | –0.026 (0.0221) –0.070, 0.017 |

^†^*p*≤0.0001

^a^Foradil^®^ Aerolizer^®^

CI, confidence interval; DPI, dry powder inhaler; FEV_1_, forced expiratory volume in 1 second; FF, formoterol fumarate; GFF, glycopyrrolate/formoterol fumarate; GP, glycopyrrolate; HR, hazard ratio; LSM, least squares mean; MDI, metered dose inhaler; mITT, modified intent-to-treat; SE, standard error
